# The Relationship Among BDNF Val66Met Polymorphism, Plasma BDNF Level, and Trait Anxiety in Chinese Patients With Panic Disorder

**DOI:** 10.3389/fpsyt.2022.932235

**Published:** 2022-06-23

**Authors:** Lijun Chu, Xia Sun, Xiaoju Jia, Dazhi Li, Ping Gao, Yong Zhang, Jie Li

**Affiliations:** Laboratory of Biological Psychiatry, Institute of Mental Health, Tianjin Anding Hospital, Mental Health Center of Tianjin Medical University, Tianjin, China

**Keywords:** panic disorder, BDNF (brain derived neurotrophic factor), gene polymorphism, BDNF plasma level, trait anxiety

## Abstract

**Background:**

Brain-derived neurotrophic factor (BDNF) is a candidate for susceptibility locus of Panic disorder (PD). However, the findings about the role of the BDNF Val66Met variant in PD were not consistent. Till now, the relationship between BDNF Val66Met polymorphism and anxiety-related traits in PD patients has been rarely explored. This study aimed to explore the relationship among BDNF Val66Met polymorphism, plasma BDNF level and anxiety-related trait in Chinese PD patients.

**Method:**

This multi-center study included 116 PD patients and 99 health controls. We detected single-nucleotide polymorphism (SNP) of BDNF rs6265 (Val66Met) and BDNF plasma level in the two groups. In addition, PD patients were administered the State-Trait Anxiety Inventory (STAI), Panic Disorder Severity Scale-Chinese Version (PDSS-CV) and Hamilton Anxiety Rating Scale (HAMA-14). Quantitative comparison of the differences of BDNF concentration among subjects with different genotypes and association between BDNF Val66Met genotype and trait anxiety were performed.

**Results:**

There were no significant differences in the genotype frequency (*p* = 0.79) or allele frequency (*p* = 0.88) between PD patients and health controls. BDNF plasma levels of PD patients were significantly lower than those in control group (*p* = 0.003). BDNF plasma levels of the Met/Met genotype were significantly lower than those of Val/Met genotype in PD patients (*p* = 0.033). PD patients carried Met/Met genotype showed significantly higher scores in STAI trait compared to those carried Val/Val genotype (*p* = 0.045) and Val/Met genotype (*p* = 0.018). STAI trait scores of PD patients with agoraphobia were significantly higher than those of patients without agoraphobia (*p* < 0.05). The ANCOVA showed that the dependent variable STAI trait score was significantly affected by factor “genotype” (Val/Val, Val/Met, Met/Met, *p* = 0.029), and covariate “agoraphobia” (*p* = 0.008). In this model, 11.5% of the variance of the STAI trait score was explained by the BDNF genotype. Contrast analysis showed STAI trait scores of Met/Met subjects were significantly higher than those of Val/Met (*p* = 0.018) and Val/Val individuals (*p* = 0.045).

**Conclusion:**

We found that anxiety trait was associated with the BDNF polymorphism in PD patients. BDNF Met/Met genotype may decrease plasma BDNF level and increase trait anxiety in panic disorder.

## Introduction

Panic disorder (PD) is characterized by recurrent and unexpected panic attacks, persistent concerns about future attacks and significant changes in behavior to avoid attacks. Panic attacks are defined as an abrupt episode of severe fear reaching a peak within several minutes, accompanied by a range of additional physiological or cognitive symptoms (1), which makes PD different from other anxiety disorders. Panic disorder is one of the most prevalent and disabling psychiatric disorders with a lifetime prevalence of 2–4% (2), while in primary care settings, the prevalence of panic syndromes is around 10% (3). The 12-month prevalence of PD is higher among women, with the female-to-male ratio as high as 2.8 (4). About 25% PD patients are comorbidity of agoraphobia, which increases severity and impairment compared with those without agoraphobia (5). Various lines of evidence suggest that both genetic and environmental factors could play important roles in contributing to PD. Family and twin studies estimate 43% of heritability for PD (6), with a relative risk of PD in proband's first-degree relatives being 8 times higher than that of healthy controls in a large-scale investigation (7). However, the genetic pathogenesis of PD remains unknown.

Brain-derived neurotrophic factor (BDNF), a neurotrophin hypothesized to limit or repair the damage caused by stress, is considered to be one plausible relevant genetic factor. BDNF plays a key role in functions and development of brain. BDNF conditional mutant mice absent of central BDNF were found to be hyperactive after exposure to stress, and had higher level of anxiety when evaluated in the light/dark exploration test (8), indicating serotonergic dysfunction might be involved in this behavioral abnormality in mutant mice. Another animal study reported that chronic administration of antidepressants promoted expression of BDNF mRNA in the rat cerebral cortex (9). Likewise, major depressed patients could display lower serum BDNF levels (10, 11), but after antidepressant treatment, the concentration of serum BDNF was higher than before (11). BDNF gene may be implicated in the putative common pathophysiology of depression and anxiety. One frequent, non-conservative polymorphism in the human BDNF gene (single nucleotide polymorphism database [dbSNP] number rs6265) has been identified, which produces a valine (Val)-to-methionine (Met) substitution at codon 66 (Val66Met). Several studies have suggested that the Met allele is related to decreased hippocampal volume and abnormal hippocampal activation in human subjects (12–14), and the hippocampus were implicated in regulating the state of mood. It could be expected that BDNF Val66Met polymorphism might influence behavior and anxiety.

The proportion of Met allele carriers of BDNF gene is significantly higher in Asians (41%) than that in Caucasians (approximately 18%), indicating an ethnic difference in the frequencies of the SNP (Val66Met) of the BDNF gene (15). Therefore, the results of BDNF Val66Met should be interpreted by racial stratification. A meta-analysis of population-based, case-control studies on BDNF Val66Met, which considered the dominant ethnicity Caucasian or Asian, suggested that the 66Met allele acted as a protective effect for substance-related disorders and exerted a risk factor for eating disorders and schizophrenia (16). Unfortunately, recent findings on relationship between the functional BDNF Val66Met and psychopathology in PD have yielded inconsistent outcomes. Some findings (17–19) reported that the BDNF gene polymorphisms are not associated with PD in neither Chinese nor Japanese population, while a meta-analysis revealed a significant association between the BDNF Val66Met and panic disorder, suggesting the Val66Met polymorphism of BDNF gene be a susceptibility factor for PD (20). The discrepancy might due to different methodologies in different studies.

According to Cloninger's theory, temperaments, including trait anxiety, are dominantly vulnerable to genetic factors. In healthy subjects, two findings showed that Met allele was associated with anxiety-related personality (21, 22), while other study evidenced that Val/Val genotype obviously dominated on anxiety trait compared to Val/Met and Met/Met genotypes (23). Another finding even reported it is BDNF serum level, not BDNF Val66Met genotype, that was correlated with anxiety personality traits in healthy people (24). In a relatively large community sample, it was found that plasma BDNF concentration was associated with depression-related personality traits in men, but not in women (25). Still in healthy participants, plasma BDNF levels were confirmed to be significantly correlated with harm avoidance, a well-known anxiety-related personality trait (26). To date, researches on the relationship between BDNF variation and trait anxiety were mostly conducted in healthy subjects. Only one study focused on the association of BDNF variation with trait anxiety in PD patients (27), however, it did not consider the factor of peripheral BDNF concentration. The changes in plasma BDNF levels could reflect variation in the release of BDNF from the brain (28). In addition, it is reported that serum and cortical BDNF levels are positively correlated (29), suggesting that it can reflect the BDNF level in the brain as well. Considering that serum BDNF is predominantly derived from clotted peripheral platelets, plasma BDNF might represent a more reliable and sensitive marker of BDNF in the brain compared to serum BDNF (30).

To the best of our knowledge, there is a lack of study on the association among BDNF Val66Met polymorphism, plasma BDNF concentration and anxiety-related personality traits in PD. We first carried out such innovative research. In this study, we examined the association between BDNF gene polymorphism and pathogenesis of panic disorder, and explored whether anxiety trait is associated with plasma BDNF level and BDNF Val66Met genotype in PD patients.

## Materials and Methods

### Participants and Study Design

Patients with PD were recruited from outpatient or inpatient department in 3 general hospitals (First Teaching Hospital of Tianjin University of Traditional Chinese Medicine, Tianjin Academy of Traditional Chinese Medicine Affiliated Hospital, and Tianjin Chest Hospital) and 1 psychiatric hospital (Tianjin Anding Hospital) in Tianjin, China.

We recruited patients based on the following inclusion criteria: (a) aged from 18 to 60 years; (b) diagnosis of PD was conducted according to the Structured Clinical Interview for the Diagnostic and Statistical Manual of Mental fifth edition (DSM-5) (SCID) criteria through a psychiatric interview; (c) total score of Hamilton Anxiety Scale (HAMA-14) ≥14; (d) total score of Panic Disorder Severity Scale–Chinese Version (PDSS-CV) ≥10. Exclusion criteria of participants included: (a) neurological diseases, severe physical diseases, comorbid other psychiatric disorder except agoraphobia. (b) use of antidepressant treatments in the past 4 weeks; (c) any physical therapy history (such as MECT and rTMS) in the past three months. Healthy controls (HCs) matched in gender, age and education levels were recruited from the local community by advertisement. The SCID was also applied to exclude lifetime prevalence of current diagnosis of both PD and other psychiatric disorders. The study protocol was approved by the Ethics Committees of the four hospitals mentioned above, and written informed consents were obtained from all participants.

### Measurements

We obtained all subjects' demographic information by a self-designed questionnaire (gender, age, marital status, education level, occupation type, past medical history, family history). PD patients were also noted their illness duration, current episode duration and whether comorbid agoraphobia.

Before study began, total two raters in Tianjin Anding Hospital who were responsible for all assessments received training program on the use of questionnaires and clinical scales. After training, repeated assessment showed that the inter-rater correlation coefficient (ICC) on the total scores of PDSS-CV and HAMA-14 were greater than 0.8. We evaluate the severity of panic symptoms and whole anxiety through the Panic Disorder Severity Scale-Chinese Version (PDSS-CV) and Hamilton Anxiety Rating Scale (HAMA-14), respectively. PDSS-CV includes seven items: panic attack frequency, distress during panic attacks, anticipatory anxiety, agoraphobic fear and avoidance, interoceptive fear and avoidance, social impairment and occupational impairment. Each item is rated on a 5-point from 0 to 4. The total score ranges from 0 to 28. The cut-off scores of this scale for severity are as follows: score 8–10 is slight; 11–13 is moderate; 14–16 is marked, and 17 and above is the most severe. The PDSS-CV has been shown excellent reliability and validity in measuring the severity of panic symptoms in Chinese patients with PD (31). HAMA-14 is one of the most widely used rating scales for the severity of anxious symptoms with good reliability and validity (32). Besides, HAMA-14 comprised 14 items, and each item is scaled by a five-point Likert ranging from 0 (absent) to 4 (severe). The total score of HAMA-14 ≥14 means definite anxiety symptoms. The Chinese version of the HAMA-14 has been demonstrated to have good reliability and validity (33). State-Trait Anxiety Inventory is a self-report assessment of state anxiety and trait anxiety, including two subscales, State-Trait Anxiety Inventory-State (STAI-S) and State-Trait Anxiety Inventory-Trait (STAI-T) (34). STAI-S and STAI-T are used for assessment the temporary condition of state anxiety and the more general and long-standing quality of trait anxiety, respectively. Each subscale consists of 20 items. The STAI-S scale is rated on 4-point likert scale (1 = not at all, 2 = somewhat, 3 = moderately and 4 = very much). Each STAI-T item is rated on a 4-point Likert scale as well (1 = rarely, 2 = sometimes, 3 = often and 4 = almost always). STAI has been shown to have good internal consistency and test-retest reliability (35).

### Genotyping and Detection of BDNF Levels

In the early morning, we took 5 mL of fasting venous blood from each patient, then spilt and stored at −80°C until later detection of BDNF plasma level and BDNF genotypes (rs6265, Val66Met). A dedicated experimental technician measured plasma concentrations of BDNF by enzyme-linked immunosorbent assay (ELISA) kit (DG10522H, Lvyuan Biotechnology) and a standard microplate reader (EL10A, BIOBASE). Two experimental technicians conducted all genotyping based on the standard procedures. The BDNF rs6265 SNP was performed via polymerase chain reaction (PCR). (The primers used for the PCR: forward 5′-ACT CTG GAG AGC GTG METT-3′ and reverse 5′-ATA CTG TCA CAC ACG CTC−3′). These samples were tested in 11 plates. The inter-and intra-assay coefficients of variation were <15%.

### Statistical Analysis

All BDNF SNPs in the present study were tested for a chi-square test-based Hardy-Weinberg equilibrium (HWE) program. First, the chi-square tests and analysis of variance (ANOVA) were used to compare demographic characteristics and plasma BDNF levels between PD group and HC group. Then, the chi-square tests were used to compare genotype distributions and allele frequency between PD patients and healthy controls. Further, the chi-square tests and ANOVA were used to compare the demographic information, clinical variables and plasma BDNF levels among three BDNF genotype subgroups. Last, we used the *t* test and ANOVA to test related variables on STAI state scores and trait scores. The effects of genotype, gender, and agoraphobia on anxiety trait and anxiety state were computed with ANCOVA. Multiple tests were adjusted using Bonferroni corrections. All tests were performed with a two-tailed type-I error rate of *p* < 0.05. Statistical calculations were carried out using SPSS 20.0.

## Results

### Demographic Characteristics and Plasma BDNF Levels Between PD Group and HC Group

As shown in Table 1, there were no significant differences in gender, age, marriage, education level, occupation between PD patients and healthy controls. For the PD patients, the duration of illness was 37.8 ± 59.6 months, and the current duration was 4.6 ± 14.1 months. Twenty (17.24%) PD patients had family history, thirty-four (29.31%) patients were first-episode patients, and thirty-three (28.45%) patients were comorbid with agoraphobia. BDNF plasma level of PD patients were significantly lower than that of healthy controls (*F* = 9.21, *p* = 0.003). In addition, a negative correlation between plasma BDNF level with age (*r* = −0.28, *p* = 0.003) was found in PD group, indicating that older PD patients had lower plasma BDNF concentrations. Zero-order correlations indicate that the other covariates including gender were not associated with plasma BDNF levels (*p* > 0.05).

**Table 1:** Demographic characteristics and plasma BDNF levels between PD group and HC group.

	**PD (*n* = 116)**	**HC (*n* = 99)**	**χ^2^/*F***	***P***
Gender (male/female)	49/67	42/57	0.24	0.62
Age (year)	47.62 ± 10.56	49.95 ± 10.43	−1.52	0.13
Marriage (married/single)	101/15	82/17	2.04	0.15
Education (L/M/H)	28/53/35	20/52/27	4.68	0.09
Occupation (labour/staff)	52/64	43/56	0.02	0.89
Plasma BDNF (ng/ml)	1.72 ± 1.02	2.11 ± 0.85	9.21	0.003*

*H, an education level of junior college or above; L, an education level lower than junior high school; M, an education level of junior high school or above but lower than junior college*.

*PD, Panic disorder; HC, Healthy control. Bold values are significant at P < 0.05*.

### Genotype and Allele Frequencies of BDNF Val66Met for all Participants

The genotype distributions of the G196A SNP (rs6265) were in agreement with the Hardy–Weinberg equilibrium in both panic disorder patients (χ^2^ = 2.84, *p* = 0.09) and healthy control group (χ^2^ = 0.44, *p* = 0.51). The genotype frequency of Val/Val (*n* = 42), Val/Met (*n* = 48), Met/Met (*n* = 26) in panic disorder group was 36.2, 41.4, and 22.4% respectively. The frequency of the alleles in panic disorder group was Val= 56.9% and Met= 43.1%. No significant differences were observed in the allele frequency or genotype frequency between PD patients and healthy controls (allele Met, *p* = 0.88, OR 1.05, 95% CI 0.72–1.54; *p* = 0.79; Table 2).

**Table 2:** Genotype distributions and allele frequency between PD patients and healthy controls.

		**Genotype**			***p^***a***^***	**Allele**		***p^***b***^***
	***n***	**Val/Val**	**Val/Met**	**Met/Met**		**Val**	**Met**	
PD	116	42 (36.2%)	48 (41.4%)	26 (22.4%)	0.79	132 (56.9%)	100 (43.1%)	0.88
HC	99	35 (35.4%)	45 (45.5%)	19 (19.2%)		115 (58.1%)	83 (41.9%)	

^a^*χ^2^ = 0.48, df = 2*.

*^b^χ^2^ = 0.02, df = 1*.

*PD, Panic disorder; HC, Healthy control*.

### Demographic Characteristics and Clinical Variables Among Different BDNF Genotype Subgroups

There were no significant differences in gender, age, marriage, education level, occupation type among BDNF genotype subgroups, neither in attack frequency, family history, comorbid agoraphobia, illness duration, current duration and STAI-S score, HAMA-14 score and PDSS-CV score (all *p* > 0.05). BDNF plasma levels and STAI-T scores among three BDNF genotype subgroups were found significantly different (*p* < 0.05). Furthermore, *post-hoc* Bonferroni comparisons showed that the BDNF plasma levels of PD patients with Met/Met genotype were significantly lower than those of Val/Met genotype (*p* = 0.033); STAI-T scores of PD patients with the Met/Met genotype were significantly higher than those of both Val/Met genotype (*p* = 0.018) and Val/Val genotype (*p* = 0.045) (Table 3).

**Table 3:** Demographic characteristics and clinical variables for PD patients separated by BDNF genotype.

	**Val/Val (*n* = 42)**	**Val/Met (*n* = 48)**	**Met/Met (*n* = 26)**	**χ^2^/*F***	***P***
Gender (male/female)	17/25	21/27	11/15	0.098	0.952
Age (year)	47.87 ± 10.00	45.02 ± 11.03	44.46 ± 11.04	1.164	0.316
Marriage (married/single)	38/4	40/8	23/3	1.073	0.585
Education (L/M/H)	12/17/13	12/23/13	4/13/9	1.920	0.750
Occupation (labour/staff)	18/24	23/25	11/15	0.318	0.853
Attack frequency (first-episode/recurrence)	11/31	17/31	6/20	1.549	0.461
Family history (yes/no)	7/35	9/39	4/22	0.149	0.928
Comorbid agoraphobia (yes/no)	10/32	13/35	10/16	1.769	0.413
Illness duration (month)	37.95 ± 69.24	34.49 ± 56.01	38.71 ± 46.52	0.055	0.946
Current duration (month)	4.16 ± 7.22	2.84 ± 3.66	3.14 ± 4.40	0.686	0.506
BDNF plasma level (ng/ml)	1.63 ± 0.96	2.02 ± 1.19	1.39 ± 0.65	3.623	0.030*
STAI-T	51.53 ± 11.41	50.58 ± 12.01	58.07 ± 8.28	4.400	0.014*
STAI-S	53.16 ± 12.88	53.05 ± 12.41	54.39 ± 12.36	0.104	0.902
HAMA-14	24.21 ± 8.86	20.81 ± 6.78	23.04 ± 8.04	1.786	0.172
PDSS-CV	15.71 ± 3.99	15.16 ± 3.30	14.71 ± 3.84	0.644	0.527

*H, an education level of junior college or above; L, an education level lower than junior high school; M, an education level of junior high school or above but lower than junior college*.

*STAI-T, State-trait Anxiety Inventory-Trait; STAI-S, State-trait Anxiety Inventory-State; HAMA-14, Hamilton Anxiety Rating Scale-14; PDSS-CV, Panic Disorder Severity Scale-Chinese Version. Bold values are significant at P < 0.05*.

### Associations Between BDNF Plasma Level, BDNF Genotypes, and Anxiety Trait

There were no significant differences in gender, marriage, education level, occupation, attack frequency, family history, comorbid agoraphobia on STAI state scores (all *p* > 0.05). The same results were observed on STAI trait scores except comorbid agoraphobia. STAI trait scores of PD patients with agoraphobia were significantly higher than those of patients without agoraphobia (57.19 ± 9.422/50.68 ± 11.565, *p* < 0.05). Age was not correlated with neither STAI state (*r* = 0.066, *p* = 0.481) nor STAI trait (*r* = 0.132 *p* = 0.158). Neither STAI state (*r* = 0.023, *p* = 0.806) nor STAI trait (*r* = −0.178, *p* = 0.055) was correlated with BDNF plasma level.

The ANCOVA showed that the dependent variable STAI trait score was significantly affected by factor “genotype” (Val/Val, Val/Met, Met/Met: *F* = 3.667, *df* = 2, *p* = 0.029), and covariate “agoraphobia” (*F* = 7.411, *df* = 1, *p* = 0.008), but not by the factor “gender” (*F* = 1.967, *df* = 1, *p* = 0.164) or “gender × genotype” interaction (*F* = 0.871, *df* = 2, *p* = 0.421). In this model, 11.5% of the variance of the STAI trait score was explained by the BDNF genotype. Contrast analysis revealed that the STAI trait scores were higher in Met/Met subjects compared to those in Val/Met (*p* = 0.018) and Val/Val individuals (*p* = 0.045). With respect to the STAI state score, ANCOVA showed no significant effects of the factor “genotype” (*F* = 0.104, *df* = 2, *p* = 0.902), “gender” (*F* = 0.686, df = 1, *p* = 0.409), or “gender × genotype” interaction (*F* = 0.169, *df* = 2, *p* = 0.845).

## Discussion

Our main findings in this current study included: (a) BDNF plasma levels of PD patients were significantly lower than those of healthy controls, and BDNF plasma levels of Met/Met genotype were significantly lower than those of Val/Met genotype in PD patients; (b) Trait anxiety in PD patients was associated with BDNF Val66Met polymorphism and comorbid agoraphobia, but not with BDNF plasma concentration; (c) No significant differences were found in BDNF allele frequencies and the BDNF genotype distributions between PD patients and health control subjects.

### Association of BDNF Plasma Level With BDNF Val66Met Polymorphism in PD

This current study revealed that there was a significant difference in plasma BDNF level between PD patients and healthy control subjects, which is consistent with previous research (36). Compared with BDNF Val carriers, the plasma BDNF levels of individuals with Met/Met genotype were the lowest in all of the PD patients. Some clinical researches also evidenced this association between the BDNF Met carrier and lower BDNF levels in the patients with major depressive disorder (37), schizophrenia, and bipolar disorder (38) respectively, suggesting the Met allele may play a key role in regulating BDNF protein expression (39). However, the results of researches in this area are inconsistent. For example, plasma BDNF concentration in Han Chinese heroin-dependent patients were not associated with BDNFVal66Met gene variants (40). And a finding with a relatively large community sample could not verify this association of plasma concentration of BDNF with Val66Met variant (25). Moreover, another study revealed significantly higher BDNF serum levels in Met carriers, compared with the Val/Val homozygotes in healthy subjects (24). The conflicting conclusions might result from different means of BDNF detection and the heterogeneity of diseases. Several technical issues influence serum or plasma BDNF levels such as clotting time, bioassays, temperature, and a second centrifugation to correct plasma levels, among others (41–43). Little data were collected on BDNF plasma concentration of different BDNF genotype subgroups in PD, more researches should be conducted.

### Association Between BDNF Val66Met Polymorphism and Anxiety Trait in PD

We found PD carriers of BDNF Met/Met were more sensitive to anxiety. This study displayed BDNF Val66Met genotype was associated with anxiety trait in PD patients, in line with a previous study (27). Yoshiaki found that the STAI trait score was highest in the Met/Met group in patients with early-onset PD, whereas the STAI trait score of the Val/Val group tended to be higher for healthy subjects. Similarly, a meta-analysis found that healthy individuals with both Met/Met and Val/Met showed a statistically significant lower neuroticism score compared to healthy people with Val/Val (44). It seemed that BDNF Met allele might be protective factor for healthy controls but a risk for PD patients on anxiety or depression personality. Some researchers believed Met/Met subgroup was more sensitive to stress, and BDNF Met-early life stress interaction predicted elevated neuroticism, higher depression and anxiety levels in PD patients (27). Another study suggested that BDNF rs6265 had a significant interaction effect with Catechol-O-methyltransferase gene (COMT) polymorphism to neuroticism and anxiety trait in PD patients, instead of healthy control subjects (45). Furthermore, a neuroimaging study has found BDNF Val66Met polymorphism is associated with white matter connectivity of the body and splenium of the corpus callosum in PD, which involved in visual memory-related processing and cognitive and affective functions (46). And these functions may be major components of PD patients with higher anxiety sensitivity (47). A recent study revealed that depending on the BDNF Val66Met polymorphism, there was an interactive genetic association between 5-HTTLPR and anxiety, and the effect of 5-HTTLPR genotype on anxiety was fully mediated by functional connectivity between the left amygdala and the right dorsolateral prefrontal cortex (48). Taken together, genetic variety could play a crucial role in trait anxiety of panic disorder. It is reported that in a sample of sixty-four healthy participants, there are differential associations between the trait anxiety measure of harm avoidance (HA) and resting regional Cerebral Blood Flow (rCBF) in BDNF Val/Val and Met carriers in several regions relevant to stress regulation brain (49). However, at the behavioral level, the relationship between BDNF Val66Met polymorphism and HA is not confirmed, thus suggesting that its effect size may be relatively small, if there is a reliable genotype–HA relationship. The effects of BDNF Val66Met polymorphism on trait anxiety in PD and the underlying mechanisms awaits further research.

### Other Findings on Trait Anxiety in PD

Considering that PD is as much as 2 times more prevalent in women than in men, a sex-specific vulnerability is involved in the etiology and/or maintenance of this disorder (50). However, we did not find sex difference on trait anxiety or state anxiety in PD. Possibly most women were during perimenopause period with average age of 46 years old. Only one study found women manifest greater trait anxiety than men did in agoraphobia patients with panic attacks (51), but other studies did not replicate this finding in panic disorder patients with agoraphobia (52, 53). It is suggested that the sex differences detected in the former study (51) may have been statistically but not clinically significant.

In addition, we found that comorbid agoraphobia is associated with anxiety trait in PD. The relationship between agoraphobia and panic disorder remains unclear. PD patients with comorbid agoraphobia were more likely to emerge serious anxiety symptom and avoidance behavior, and agoraphobia was considered to result from recurrent panic attacks (54). A recent study also showed that patients with PDA (panic disorder with agoraphobia) had severer anxiety symptom and higher anxiety trait than those with PD alone (55). PDA patients were reported greater self-criticism and fatalism (56) and higher severity of fear of bodily sensation (57) than those with PD alone. Here, we highlight the importance of recognizing comorbid agoraphobia with PD.

### Association Between BDNF Val66Met Polymorphism and Panic Disorder

Our findings found no association between BDNF Val66Met polymorphism and panic disorder, which was consistent with previous findings in Japanese and Chinese PD patients (17, 19, 27). This result indicated that BDNF rs6265 may not contribute to PD susceptibility. Reasons for the lack of association reported in various studies might partly be explained by the role of BDNF in conjunction with other “hypothetical” genes (58, 59). In addition, PD patients with the G-C haplotype for 196G/A (rs6265) and 11757G/C (rs16917204) may be more susceptible in the development of PD (60). Therefore, more researches with high-quality design, large samples are needed to explore the association between BDNF Val66Met variant and panic disorder.

## Limitation

The limitations of our study come as follows. First, STAI score of healthy controls in different BDNF Val66Met genotypes were not evaluated. Hence, we cannot distinguish the levels of anxiety between PD patients and healthy controls. The lack of relevant data collection is a methodological limitation in this study. Second, the sample size was relatively small. Therefore, the effect of gender on anxiety trait in PD may be underestimated. Third, we only used STAI to test individual's anxiety trait, more anxiety-related traits could be evaluated by other scales such as the Harm Avoidance (HA) scale of Tridimensional Personality Questionnaire (TPQ), which may enhance credibility of the study. Moreover, the measure of trait anxiety, using self-administered questionnaires, may be susceptible to reporting bias. Fourth, this is a cross-sectional design, long-term psychopathology may not be reflected by a single point assessment. Last but not least, the interaction of gene plus gene should be considered, such as BDNF gene and COMT gene. And haplotype for the three SNPs (rs6265-rs16917204- rs56164415) in gene BDNF should be taken into consideration to explore BDNF gene polymorphism and anxiety trait.

## Conclusions

In summary, we first explored the relationship among BDNF Val66Met polymorphism, plasma BDNF level and anxiety trait in PD patients. We found that BDNF Val66Met genotype is associated with anxiety trait in PD patients. BDNF Met/Met genotype may decrease plasma BDNF level and increase trait anxiety in panic disorder. However, due to the small sample and other limitations, these results should be replicated in a larger sample of high-quality study design.

## Data Availability Statement

The original contributions presented in the study are included in the article/supplementary materials, further inquiries can be directed to the corresponding authors.

## Ethics Statement

The studies involving human participants were reviewed and approved by the Ethics Committees of Tianjin Anding Hospital. The patients/participants provided their written informed consent to participate in this study.

## Author Contributions

LC, JL, and YZ designed the study and obtained the data. XS undertook the analysis supervised by JL. LC and XS wrote the manuscript. XJ, DL, and PG performed the survey. All authors read the final manuscript and agreed with the text.

## Funding

This work was supported by Health Science and Technology Project of Tianjin (Grant No: TJWJ2021QN065).

## Conflict of Interest

The authors declare that the research was conducted in the absence of any commercial or financial relationships that could be construed as a potential conflict of interest.

## Publisher's Note

All claims expressed in this article are solely those of the authors and do not necessarily represent those of their affiliated organizations, or those of the publisher, the editors and the reviewers. Any product that may be evaluated in this article, or claim that may be made by its manufacturer, is not guaranteed or endorsed by the publisher.
